# Supplementary material for: Multiplexed Human Gene Expression Analysis Reveals a Central Role of the TLR/mTOR/PPARγ and NFkB Axes in Burn and Inhalation Injury-Induced Changes in Systemic Immunometabolism and Long-Term Patient Outcomes
Source: Int J Mol Sci. 2022 Aug 20;23(16):9418. doi: 10.3390/ijms23169418 (PMC9409318; doi:10.3390/ijms23169418)
Supplement: Supplementary file 1 [file ijms-23-09418-s001.zip › ijms-1821226-supplementary/Supplemental Figures_new.pdf]

mTOR Signaling *versus* healthy

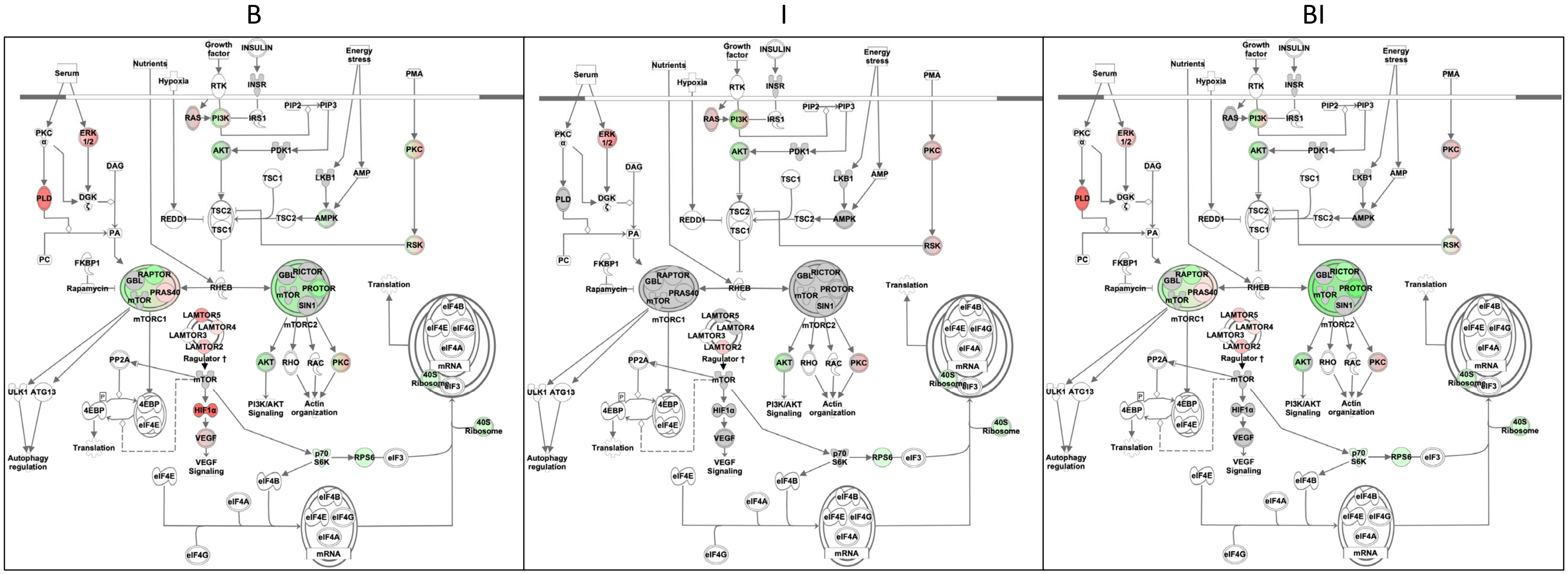

Figure S1

TLR Signaling *versus* healthy

B I BI

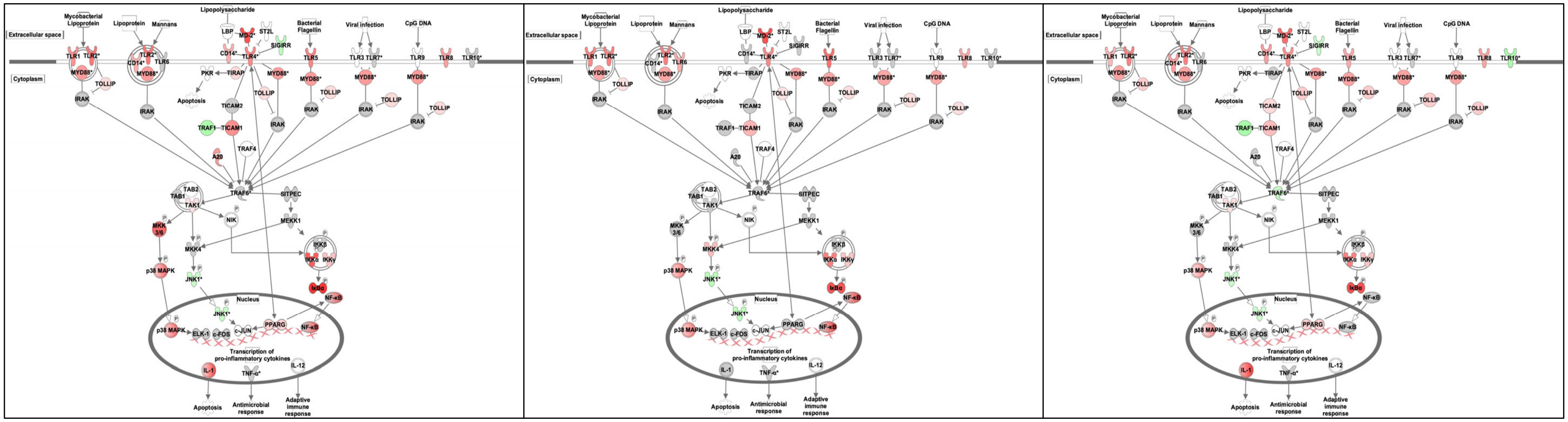

Figure S2

NF-kB Signaling *versus* healthy

B I BI

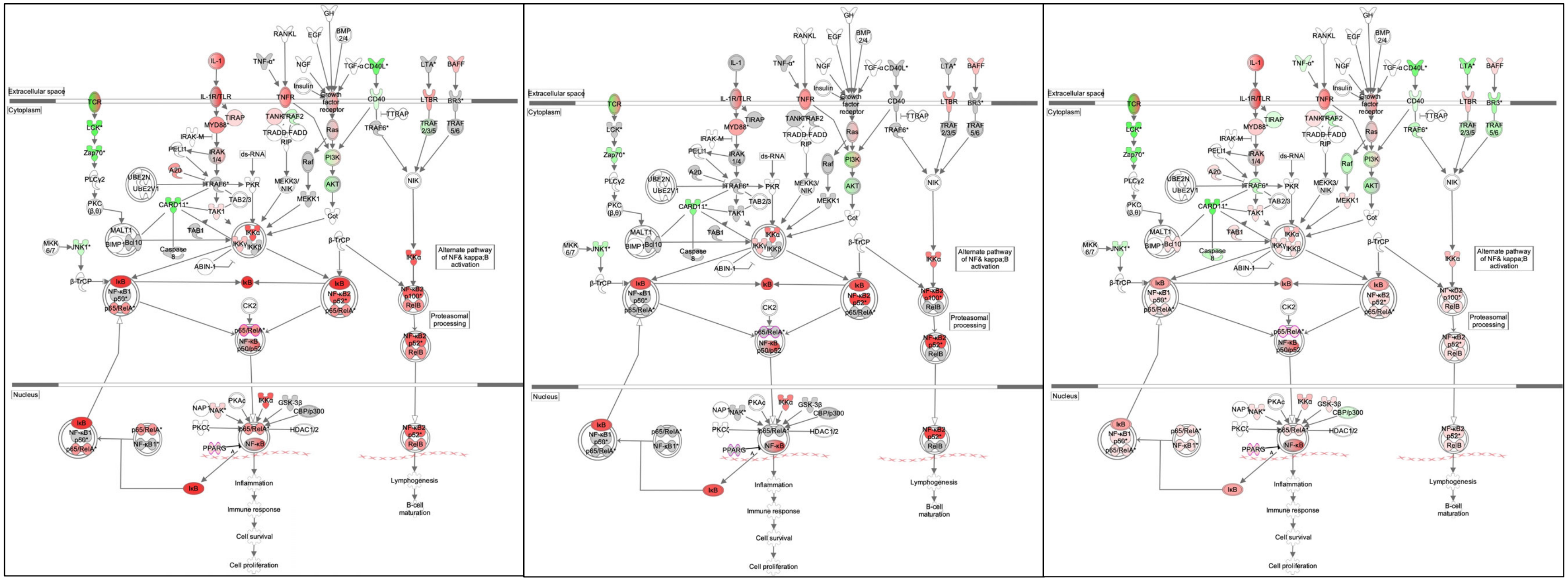

Figure S3

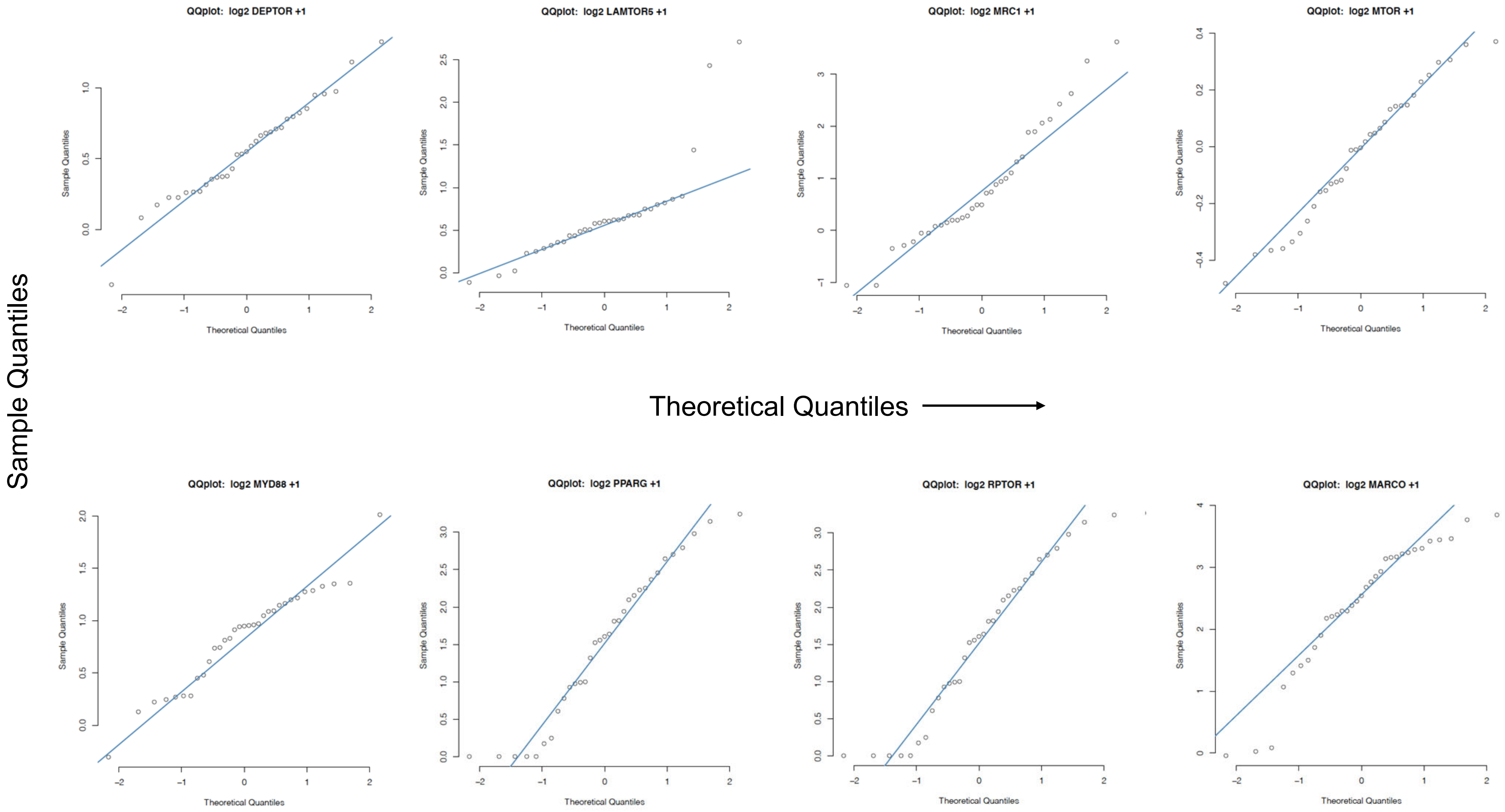
QQplot: log2 DEPTOR +1
QQplot: log2 LAMTOR5 +1
QQplot: log2 MRC1 +1
QQplot: log2 MTOR +1
QQplot: log2 MYD88 +1
QQplot: log2 PPARG +1
QQplot: log2 RPTOR +1
QQplot: log2 MARCO +1
Sample Quantiles
Theoretical Quantiles

Figure S4
